# Measuring the Business Cycle Chronology with a Novel Business Cycle Indicator for Germany

**DOI:** 10.1007/s41549-021-00054-6

**Published:** 2021-04-09

**Authors:** Agnieszka Gehringer, Thomas Mayer

**Affiliations:** 1 Flossbach von Storch Research Institute, Cologne University of Applied Sciences, Cologne, Germany; 2 Flossbach von Storch Research Institute, University of Witten-Herdecke, Witten, Germany

**Keywords:** Business cycle dating, Recession, Principal component analysis, German economy, C14, C82, E32, E65

## Abstract

This paper introduces a Business Cycle Indicator to compile a transparent and reliable chronology of past business cycle turning points for Germany. The Indicator is derived applying the statistical method of Principal Component Analysis, based on information from 20 economic time series. In this way, the Business Cycle Indicator grasps the development of the broader economic activity and has several advantages over a business cycle assessment based on quarterly series of Gross Domestic Product.

## Introduction

The need to reliably measure the business cycle turning points is well-grounded in the interest to understand the causes and consequences of fluctuations in economic activity. A deep understanding of cyclical movements is crucial for a correct assessment of the state of economic momentum and for a better understanding of future developments (Anas et al., 2007; Mazzi & Scocco, 2003). However, the interest in cyclical fluctuations goes beyond academic analysis and is also relevant for economic policy, which looks for ways and instruments to smooth economic cycles. A similar motivation stood at the origins of the early national accounting framework developed by Colin Clark and Simon Kuznets in the 1930s (Clark, 1932; Kuznets, 1937). It also has its roots in the pioneering analysis of the business cycle and economic crises (Berge & Jordà, 2011).

Since 1979, the National Bureau of Economic Research (NBER) has documented and officially announced the turning points in the business cycle of the US economy.^1^ Based on a thorough analysis of macro indicators of real economic activity, the NBER retrospectively determines the dates of troughs as the starting date of the expansion, and the dates of peaks as the starting date of the recession.^2^ Specifically, according to the NBER definition:

A recession is a significant decline in economic activity spread across the economy, lasting more than a few months, normally visible in production, employment, real income, and other indicators. A recession begins when the economy reaches a peak of activity and ends when the economy reaches its trough. Between trough and peak, the economy is in an expansion.^3^

A similar procedure does not exist for Germany.^4^ The German Council of Economic Experts (Sachverständigenrat zur Beurteilung der gesamtwirtschaftlichen Entwicklung) introduced in 2009 a recession criterium, according to which “a recession occurs when a decline in the relative output gap by at least two thirds of the respective potential growth rate is accompanied by a currently negative output gap.” However, it is not specified on which time period the criterion should be applied. Moreover, by its nature, the output gap is an unobservable concept, which must be estimated. However, the methodology underlying the estimations of the concept has been often criticized (Brooks & Basile, 2019; Kuusi, 2018; Tooze, 2019). Finally, beyond this definition of the recession by the German Council of Economic Experts, there is no systematic and transparent procedure to assess the historical chronology of the turning points in the business cycle in Germany. The customary approach followed by experts and observers is to look at the development of quarter-over-quarter growth rates of real GDP. If at least two consecutive quarters of contraction occur, the economy is said to enter a “technical recession”.^5^

However, this definition has at least two drawbacks. First, by focusing on two-quarters only, it disregards the trend development of the economy.^6^ Second, GDP data are only available on a quarterly basis, with the flash estimates often subject to subsequent revisions. This implies serious delays in the business cycle observation, which is challenging especially for policymaking (Galli, 2018).

To counter these limitations, this paper introduces the Business Cycle Indicator (BCI) for Germany, offering a timely measure of business cycle developments and a reliable basis for monthly business cycle dating, which have been missing so far (Carstensen et al., 2020). The only available monthly chronology disseminated by the Economic Cycle Research Institute (ECRI) lacks the important methodological background needed to assess its reliability.^7^

Based on the novel BCI, our main objective is in dating cyclical developments for the German economy. Our methodology follows the spirit of the NBER’s approach in terms of the identification of the turning points of the business cycle. However, in contrast to the NBER, we place less emphasis on discretion, given that we derive and implement a single, transparent and quantitative indicator to assess the evolution of the business cycle. In doing so, we apply principal component analysis (PCA) to 20 economic time series and arrive at a single indicator, which we then use in a simple graphical inspection to arrive at a comprehensive dating of the business cycle in Germany. To corroborate our qualitative approach we apply the standard algorithm method by Bry and Boschan (1971) as a sensitivity check.

Our BCI has three main advantages for the analysis of the business cycle compared to the traditional methods: First, by relying on the information obtained directly from a broad list of economic activity data available on a monthly basis, it can more timely than real GDP and reliably identify business cycles peaks and troughs. Second, as it is based on monthly data it can more precisely time turning points of the business cycle than it is possible based on quarterly observations. Related to this, and thirdly, the BCI permits us to gain a more detailed view on the developments between peaks and troughs and thus to better understand the stylized facts of the business cycle in Germany.

In the remaining part of the paper we first place our contribution within the relevant literature (Sect. 2). We then present in more detail our methodology and data (Sect. 3), discuss the results (Sect. 4) and check for robustness of the BCI and of the business cycle dating approach (Sect. 5). In the last section we provide concluding remarks to our analysis.

## Literature Review

There is an extensive literature on the measurement and analysis of the business cycle development to which our paper closely refers. Related to our objective to identify turning points, we align with the seminal contribution of Burns and Mitchell (1946), who based their identification approach on the analysis of changes in the absolute level of relevant economic indicators.

Among the other related papers, Anas et al. (2007), Artis et al. (2004a) as well as Krolzig and Toro (2005) deal with the measurement of the European business cycle. In particular, Artis et al. (2004b) apply a non-parametric algorithm, various assessment criteria, and “expert judgements” with the final aim to measure the degree of diffusion and synchronization of the cycles among the euro area countries. However, they do not provide details on dating for single euro are members. Against this, Artis et al. (2004a) identify the business cycle duration and amplitude for European countries taken as a group and for individual countries, inter alia Germany. Moreover, Krolzig and Toro (2005) compare the classical [in the spirit of Burns and Mitchell (1946)] and modern methods of business cycle measurement (a Markov-switching time series model as proposed by Hamilton (1989)) and confirm a high degree of similarity between the two approaches in cycle dating.

From the point of view of the statistical method employed to identify the turning points of the business cycle, we refer to the pioneering work laid down by Burns and Mitchell (1946) and subsequently Bry and Boschan (1971) at the National Bureau of Economic Analysis. Burns and Mitchell (1946) define turning points as points in time when a cross-section of economic indicators change direction—from positive to negative, or vice versa. Along this line of analysis, Bry and Boschan (1971) developed an algorithm, which is very intuitive as it applies faithfully the NBER’s recession definition, as proposed by Burns and Mitchell (1946). It takes the raw series—seasonally adjusted—and searches for local minima and maxima in these series. Within one cycle, a local minimum (the trough) is followed by a local maximum (the peak). The period between trough and peak is an expansion, and that between peak and trough a recession. A completed cycle, which is the interval from the initial to the final trough, is conditioned on a minimum duration of 15 months. Both recessions and expansions should have a minimum duration of six months. Finally, the peak-trough amplitude is the difference between the level of the time series at adjacent peaks and troughs.

We follow the NBER’s methodology of dating of business cycles in principle. But we identify the turning points based on our single indicator, rather than on a cross-section of economic variables. In this way, we overcome the lack of measurement precision of aggregate economic activity as pointed out by Harding and Pagan (2002). However, whereas Harding and Pagan (2002) claim that only economic output—as measured in terms of GDP—can be viewed as a relevant measure of the business cycle, we recognize that economic activity is a much broader and more abstract concept than output and is better described as well as measured in a multidimensional framework.

It is worth noting that the method by Bry and Boschan (1971) is simple to apply and to reproduce. At the same time, it can be implemented only on individual time series. This may be a limitation, considering that economic fluctuations are a result of co-movements of multiple contemporaneous processes. For that reason, more structural methods were developed, following Hamilton’s (1989) application of the Markov-switching autoregressive (MS-AR) time series model to measure the US business cycle. Similarly, like the Bry and Boschan (1971) method, the univariate MS-AR models as originally proposed by Hamilton (1989) are unable to reflect co-movements among time series. For that reasons, later researchers moved to model vectors of time series, more accurately capturing the fact that business cycles often derive from a common feature of multiple simultaneous variables.

Large-scale dynamic factor models were developed to construct coincident indexes, taking advantage of the increasing availability of multiple data sources (“big data”) (for instance, Forni et al., 1999, Altissimo et al., 2001, Watson, 2003, Chauvet & Hamilton, 2006, and more recently Galli, 2018).^8^ With hundreds of series, these models can be used to establish a turning point chronology by applying parametric or non-parametric procedures. As such, these procedures can extract much more detailed information compared to approaches—like ours—which use a limited number of economic series. At the same time, these procedures are flexible enough to account for certain non-linearities of the cycle, such as different durations, amplitudes, and cumulative movements of its phases. But this may also be a disadvantage compared to qualitative procedures based on graphical inspection, which normally do not impose any a-priori rule on the shape of business cycle phases. The inherent uncertainty underlying the assessment of the business cycle is deemed lower when qualitative rather than quantitative approaches are used (Chauvet & Hamilton, 2006).

We recognize the need to take a systematic approach to the investigation of the business cycle in Germany, but at the same time we aim to avoid the aforementioned limitations of purely quantitative approaches. The systematic nature of the analysis is preserved by applying the principal component analysis (PCA) to 20 economic time series. At the same time, the rigidity of quantitative approaches to business cycle dating is avoided by using a simple graphical inspection. Specifically, based on PCA we arrive at a single indicator encapsulating the business cycle in Germany. With this indicator at hand, our main aim is to identify the turning points in the business cycle, following the approach adopted by the NBER. However, to control for the risk that the qualitative assessment of the business cycle is at variance with the quantitative approach, we check our baseline results by applying the standard algorithm method of Bry and Boschan (1971) as a sensitivity check.

Finally, we deliberately leave the growth cycle turning points dating literature aside and focus on the classical approach only.^9^ The reasons for this are that, firstly, we aim at staying as close to the widely accepted NBER approach as possible and, secondly, we want to avoid the disadvantage of the growth cycle approach, due to the fact that it is dependent on the underlying detrending method, which is empirically a disputable issue (Anas et al., 2008; Canova, 1994).

## Methodology

The NBER’s Business Cycle Committee decides on the turning points (trough and peak) in the US business cycle several quarters after the passing of the turning points. The Committee waits until a sufficient amount of data is available to avoid the need for major ex-post revisions. The idea is to infer from these data on real GDP (and real Gross Domestic Income, GDI), which the Committee regards as the best single measure of aggregate economic activity. By combining monthly data with GDP data, it is not only possible to better assess lasting turning points of GDP, but also to time these turning points more precisely.

For instance, in September 2010, based on real GDP and GDI, which reached their lows in the second quarter of 2009, the Committee concluded that the trough occurred in mid-2009. With the help of several monthly indicators (estimated monthly GDP, manufacturing and trade sales, industrial production, real personal income less transfers and labor market indicators), the Committee then was able to identify June as the month of the trough. Similarly, for the previous turning points the NBER announced in April 1991 that a peak in the US cycle occurred in July 1990, and in December 1992 that there was a trough in March 1991. The most recent announcement was an exception to the rule. On June 8, 2020, the Committee determined that a peak in monthly economic activity in the USA occurred only four months earlier, in February 2020, marking the end of the 128-months long expansion—the longest in the history of US business cycles dating.^10^

Following the NBER’s approach, we analyze a wide range of economic indicators to better capture the overall development of the German economy. However, we depart from their approach in two important respects. First, we avoid looking at quarterly GDP or GDI data, and instead look at a broader list of monthly indicators, also with data covering only a part of the economy. Second, based on these monthly indicators, we use principal component analysis (PCA) to derive a single and reliable Business Cycle Indicator for Germany to capture swings of the business cycle. These methodological innovations with respect to the NBER’s approach are important as they allow us to avoid the issue of repeated data revisions, which is typical for GDP figures. Indeed, as new available surveys come in and methodological improvements are integrated, GDP series need to be revised. This causes sometimes substantial delays in announcing the turning points of the business cycle.^11^ Accordingly, using other economic indicators than GDP should reduce the problems caused by the delay (Anas et al., 2007). Moreover, by applying PCA and deriving the single BCI, we base our judgement concerning the turning points on a more transparent and comprehensive procedure.

PCA, and more generally, factor models are used in frameworks with a large number of closely related variables where multicollinearity is a risk. The aim is to reduce dimensionality of the system by identifying the most important influences from these variables. This is achieved by exploiting the correlations among the regressors to reduce their number, but at the same time retaining as much of the information in the original predictors as possible (Stock & Watson, 2020). Accordingly, the principal components maximize the variance of the linear combination of the variables.

Analytically, if there are *n* explanatory, closely related variables in the regression model, PCA transforms them into *n* uncorrelated new variables (principal components), of the form:

1 $$\begin{array}{*{20}c} {p_{1} = \alpha _{{11}} x_{1} + \alpha _{{12}} x_{2} + \cdots + \alpha _{{1n}} x_{n} } \\ {p_{2} = \alpha _{{21}} x_{1} + \alpha _{{22}} x_{2} + \cdots + \alpha _{{2n}} x_{n} } \\ \cdots \\ {p_{n} = \alpha _{{n1}} x_{1} + \alpha _{{n2}} x_{2} + \cdots + \alpha _{{nn}} x_{n} } \\ \end{array}$$

where *x*_*j*_*,* and *p*_*i*_ (with *i*, *j* = 1, …, *n*) are the original explanatory variables and the newly estimated principal components, respectively, and *α*_*ij*_ are estimation coefficients (so called factor loadings) on the *j*th explanatory variable in the *i*th principal component. It is required that the sum of the squares of the coefficients for each component is one:

2 $$\mathop \sum \limits_{j = 1}^{n} \alpha_{ij}^{2} = 1\quad \forall i = 1, \ldots ,n$$

The principal components are derived in descending order of importance. Moreover, in the case of collinearity of the original variables, the first components will account for much of the variation, whereas the last few principal components will account for little variation and can be discarded. The stronger the correlation between the original variables, the higher is the explanatory power of the first principal components.

To validate PCA, the so-called Kaiser-Mexer-Olkin’s (KMO) measure of sampling adequacy can be calculated. KMO takes values between 0 and 1, with relatively high values suggesting that variables have sufficient in common to warrant a PCA. Small KMO values suggest that the sample is insufficiently adequate to apply a PCA.

A potential issue within the framework of the PCA may occur when the underlying time series are affected by exogenous trends and have complex structures, resulting in non-stationary series (Schmitt et al., 2013; Zhao & Shang, 2016). The presence of non-stationarity, which may reflect a persistent trend in the series, could increase the value of the variance that is maximized for every principal component, but at the same time deliver poor information by the component (Zhao & Shang, 2016). Specifically, under non-stationarity, the PCA could result in a few components assigning similar factor loadings to all variables (Lansangan & Barrios, 2009).

We therefore analyse the time-series properties of our data first. If they are non-stationary, we perform the PCA analysis on first-differenced data and recalculate our Business Cycle Indicator, which we then compare with its baseline estimate. Given that a trend is the most important driver of non-stationarity, this exercise should easily reveal how much of a problem the PCA with non-stationary data is in our framework.

In our PCA exercise, we use 20 economic indicators for which we can rely on monthly observations and which together cover the entire breadth of activity in the economy (Table 1). Given that we use our BCI for the inspection of the past business cycle performance, we focus on hard data only, which deem to reflect the actual economic situation of the real economy. Accordingly, our data set does not include financial series, like the stock market index, interest rates or exchange rates, which undeniably might send important cyclical signals, but by their nature are rather volatile around the cycle. This could contribute to an undesired noisiness of the incoming signals. We also do not consider survey information, like the purchasing manager index or different sentiment or confidence indicators, given that they often send premature or exaggerated signals on the cyclical state of the economy.

**Table 1:** Monthly data used in the Principal Component Analysis

Indicator	Description of raw series and starting date
Sales overall, of which	
Manufacturing	Constant prices, Index, 2015 = 100, since Jan. 1991
Intermediate goods	Constant prices, Index, 2015 = 100, since Jan. 1991
Capital goods	Constant prices, Index, 2015 = 100, since Jan. 1991
Cars & car parts	EUR, since Jan. 1991
Sales domestic, of which:	
Manufacturing	Constant prices, Index, 2015 = 100, since Jan. 1991
Intermediate goods	Constant prices, Index, 2015 = 100, since Jan. 1991
Capital goods	Constant prices, Index, 2015 = 100, since Jan. 1991
Cars & car parts	EUR, since Jan. 1991
Retail trade	Constant prices, Index, 2015 = 100, since Jan. 1991
Wholesale trade	Constant prices, Index, 2015 = 100, since Jan. 1994
Employment	No. of persons, domestic concept, since Jan. 1991
Industrial production, of which	
Intermediate goods	Constant prices, Index, 2015 = 100, since Jan. 1991
Capital goods	Constant prices, Index, 2015 = 100, since Jan. 1991
Consumer goods	Constant prices, Index, 2015 = 100, since Jan. 1991
Electricity, gas, steam & air-cond	Constant prices, Index, 2015 = 100, since Jan. 1991
Vehicle registration, trucks	No., since Jan. 1991
Vehicle turnover	Constant prices, Index, 2015 = 100, since Jan. 1994
International trade, of which	
Import volume	Index of unit values, 2010 = 100, since Jan. 2008
Export volume	Index of unit values, 2010 = 100, since Jan. 2008
Service trade, turnover	Constant prices, Index 2015 = 100, since Jan. 1994

Related to this, we use the largest possible set of hard-data indicators. Nevertheless, our data coverage remains narrow compared with analyses applying large-scale dynamic factor models, like the one by Galli (2018). However, more recent contributions in this field tend to indicate that smaller sets of indicators capture more reliably business cycle dynamics than larger sets do (Aastveit et al., 2016; Camacho & Martinez-Martin, 2015; Carstensen et al., 2020).

All raw series are calendar and seasonally adjusted. We additionally use smoothed data, which are calculated as centered moving averages over one-year periods. Since PCA is scale sensitive, we index all time series to January 2019 = 100.

The longest data series are available back to 1991, but some series are available only starting in 2008 (international trade data). For this reason, the workable version of our monthly BCI, which we will update on a regular basis, is available from January 2008. However, to validate our model prior to 2008 we compare the performance of the Indicator with quarterly real GDP data back to 1991.

The use of PCA in the field of business cycle analysis builds on the pioneering works of Stock and Watson (1988, 2002), Harvey (1990), Harvey and Jäger (1993), Harvey and Trimbur (2003), and Forni et al. (1999). These authors developed the formal approach for the derivation and estimation of common cycles, based on the idea that the business cycle is the common factor in the economy.^12^ Specifically, the crucial contribution of Stock and Watson (1988) was to show that a common component is a fundamental aspect of the underlying dynamics in any economic system. The validity of their finding was confirmed subsequently, using other, more sophisticated methods, like unobserved components models (Harvey, 1990, Harvey and Jäger 1993, Harvey and Trimbur 2003) and large-scale dynamic factor models (Forni et al. 1999; Watson, 2003).

To our knowledge, PCA has so far not been applied to the German business cycle. Although we adopt the graphical inspection as our main approach, we make sure that the procedure is transparent and understandable. Moreover, we compare the results from our qualitative approach with the ones we obtain from a recognition pattern algorithm (Bry & Boschan, 1971).

## Results

Our PCA estimates show that the first principal component is responsible for almost 74% of variation in our set of explanatory variables. In Table 2, we report the factor loadings corresponding to each variable from the first principal component. Each of the remaining 18 principal components is negligible, since their individual contribution to the overall sample variation is under 1%. Hence, we construct our BCI based on the first principal component.

**Table 2:** Factor loading of the first principal component

Indicator	Factor loading
Sales overall, of which	
Manufacturing	0.2666
Intermediate goods	0.2487
Capital goods	0.2661
Cars & car parts	0.2570
Sales domestic, of which	
Manufacturing	0.2117
Intermediate goods	0.1989
Capital goods	0.2455
Cars & car parts	0.2439
Retail trade	0.1644
Wholesale trade	0.1982
Employment	0.1907
Industrial production, of which	
Intermediate goods	0.2513
Capital goods	0.2558
Consumer goods	0.2390
Electricity, gas, steam & air-cond	0.0552
Vehicle registration, trucks	0.2421
Vehicle turnover	0.2049
International trade, of which	
Import volume	0.2404
Export volume	0.2525
Service trade, turnover	0.1114

Since the squares of the estimated coefficients for a principal component add up to one, we use the coefficients of the first principal component to weigh the respective explanatory variables. Thus, the BCI is a weighted average of our monthly indicators.

**Fig. 1:**
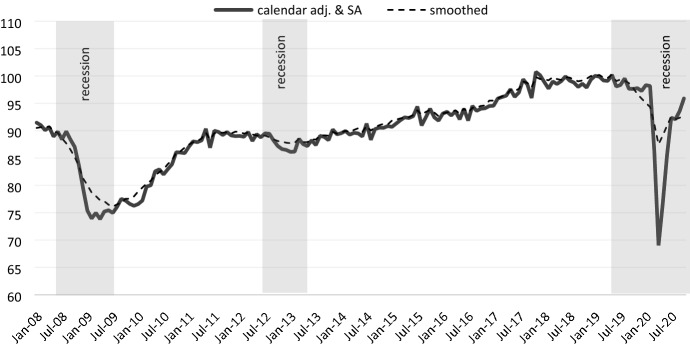
Business Cycle Indicator for Germany, Index (Jan 2019 = 100). Source: Own calculations Flossbach von Storch Research Institute/Macrobond

Figure 1 shows the monthly BCI both as calendar and seasonally adjusted (solid line) and smoothed series (dotted line). The BCI stagnated from the second quarter of 2018 and started to decline in March 2019. Hence, we identified this month as the starting point of the most recent recession. The declines were sometimes substantial, as was the case in June, September and eventually December of 2019. The few months with positive growth during 2019 could not compensate for these declines. This development is much in line with the negative growth rate of real GDP in the second quarter of 2019 and with very tepid growth in the final two quarters of the year (0.2% in the third and 0.0% in the fourth quarter). Albeit not a forecasting instrument, the BCI could have anticipated this.

The overall subdued development of the economy during 2019 was subsequently negatively impacted by the pandemic, which drove the economy into a much deeper recession starting in March 2020. It is thus important to note that since February 2019 we have experienced two overlapping patterns, which we, however, are unable to distinguish with our instrument. The first recession period, between February 2019 and February 2020, is unrelated to the pandemic and has more to do with the negative impact of the ongoing trade disputes and deglobalization tendencies. The second period is unambiguously related to the pandemic.

Looking at the past development of the BCI around the Great Financial Crisis, it emerges that it would have signaled in a timely way the subsequent recession in Germany. The Indicator reached the peak in May 2008, which indicates the start of the recession. It then reached the trough in February 2009, which marks the end of the recession and the starting point of the next business cycle.

Another visible, although rather mild economic recession occurred at the time of the European sovereign debt crisis in mid-2012. The Indicator reached the peak in March 2012 (beginning of the recession) and the trough in January 2013 (end of the recession).

**Fig. 2:**
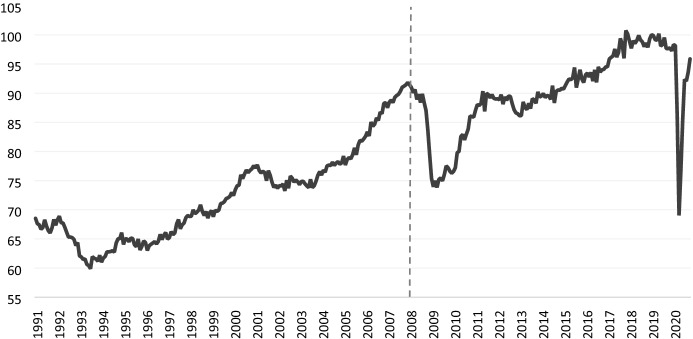
Monthly Business Cycle Indicator for Germany for the period 1991–2020, Index (Jan 2019 = 100). Source: Own calculations Flossbach von Storch Research Institute/Macrobond

As already discussed above, our operational version of the Indicator, which we will continue to update on a monthly basis, is available since 2008. To determine a historical record of recessions of the German economy since 1991 on a monthly basis, we calculate a monthly series for the BCI for the period 1991–2007 based on estimates with the restricted set of data as explained in the previous section and splice this series with the series calculated with the full set of data as of 2008. Figure 2 shows the time series of the Indicator, with the structural break due to the enlarged set of series marked between December 2007 and January 2008.

Based on this combined monthly series of the BCI shown in Fig. 2, we inspect the Indicator to determine the monthly dates of the turning points in the business cycle starting in 1991 (see Table 3). A recession from peak to trough is identified when the BCI shows a sustained decline followed by a sustained recovery.

**Table 3:** Turning points in the business cycle and the duration of contractions and expansions in Germany

Peak	Trough	Duration in months	
		Contraction (recession)—peak to trough -	Expansion—previous trough to peak
February 1991 (Q1)	January 1994 (Q1)	36	–
January 1995 (Q1)	February 1996 (Q1)	13	12
April 1998 (Q2)	September 1998 (Q3)	6	25
January 2001 (Q1)	August 2003 (Q3)	32	27
May 2008 (Q2)	February 2009 (Q1)	12	56
April 2012 (Q2)	December 2012 (Q4)	9	36
February 2019 (Q1)	?	?	62

Source: Own elaborations Flossbach von Storch Research Institute based on the BCI, Macrobond

In our dating exercise, we implement raw BCI series, rather than the smoothed one. In doing so, we aim at avoiding the lag in inspecting economic activity, which typically exists when using the smoothed series (Zhao, 2020).^13^

Comparing our business cycle dating with the one by the ECRI, we observe a close correspondence with the major past recessions. There is a perfect match in the dates of the 2001–2003 recession. For the Great Financial Crisis, the ECRI identified the peak in April 2008 and the following trough in January 2009, which is one month earlier than our Indicator. At the same time, based on our dating procedure, we could identify a recession event around the European sovereign debt crisis, which is not detected through the ECRI’s business cycle chronology.

## Robustness Analysis

A convincing validation of the Indicator requires that its long-term past performance mirrors the development of real GDP. This is especially important since real GDP is customarily the reference indicator for policy makers and practitioners to assess the business cycle dynamics and recessions.

The preferred approach would be to estimate a series of monthly GDP, in line with the approach followed by Stock and Watson at the NBER Business Cycle Dating Committee, and then compare our Indicator with this monthly series. However, due to data limitations we are unable to adopt this method. Hence, to check the robustness of our Indicator, we followed a different approach based on the so called mixed-data sampling (MIDAS), pioneered by Ghysels et al. (2002). Within this method, the dependent variable is recorded at a lower frequency (e.g. quarterly) than the independent variables (e.g. monthly).^14^

**Table 4:** OLS estimation results of real GDP and the first three principal components (PC)

	Specification (1)	Specification (2)
*PC 1*	0.780*** (0.017)	0.951*** (0.039)
*PC 2*	–	− 1.012*** (0.141)
*PC 3*	–	0.349*** (0.048)
R-squared adj	0.952	0.967

***, ** and * show 1%, 5% and 10% significance levels. Standard errors are in parentheses. Estimations are performed with robust standard errors

Source: Own estimations Flossbach von Storch Research Institute

The regression to be estimated has the following general form:

3 $${y}_{T}={\beta }_{0}+{\beta }_{1}{p}_{1t}+\dots +{\beta }_{r}{p}_{rt}+{\varepsilon }_{t}$$

where *y*_*t*_ is real GDP in quarter *T*, *β*_*m*_ (with *m* = 1, …, r) are the estimation coefficients, *p*_*mt*_ are the first *r* (0 < *r* < *n*) principal components deemed sufficiently useful in explaining the variation of *n* original variables. These series of principal components are recorded at monthly frequency. Finally, ${\varepsilon }_{t}$ is an idiosyncratic error term. The estimation sample spans between 1991 and the end of 2020.

From the PCA on the set of indicators used to derive the BCI, we could confirm that the first principal component explains almost 70% of the sample variation. The second principal component adds 19% and the third one 4%. Thus, cumulatively, the first three components are responsible for almost 93% of the sample variation. Thus, we based our regression on these three first components.

The results of the estimations in Table 4 show that the first principal component explains 95% of the variability in real GDP (R-squared adjusted in the first specification). Moreover, the addition of the second and third principal component improves further the goodness of fit of the regression by two percentage points. Altogether, the BCI explains most of the variation observed over time in real GDP data.^15^

**Fig. 3:**
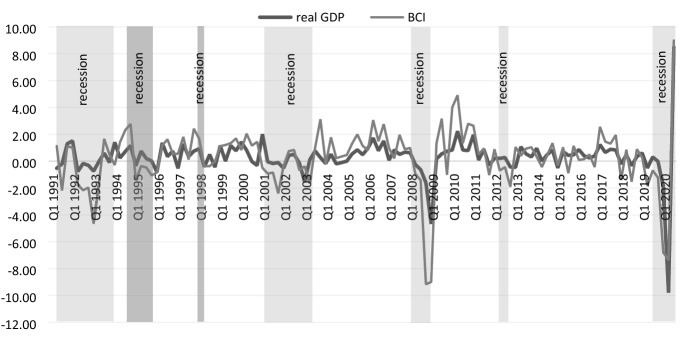
Quarter over quarter growth rates of the Business Cycle Indicator and real GDP in Germany. Source: Own calculations Flossbach von Storch Research Institute/Macrobond

To illustrate the relationship between the BCI and the real GDP data, we re-estimated the Principal Component Analysis for our sample of indicators on a quarterly basis for which data are available since 1991 and plotted this quarterly BCI together with real GDP. Figure 3 shows the quarter-over-quarter growth rates of both series. Although the Indicator overestimates the growth rates at the extremes (both positive and negative), it tracks with sufficient precision real GDP growth.

We find a general concordance between the technical recession definition based on real GDP and the recession determination based on our quarterly series of the BCI, as shown by the shaded recession areas in Fig. 3 and as listed in Table 3 above.^16^ There is a perfect overlapping between the two recession definitions for the recession 2008/2009. Also, the starting quarter of the most recent recession episode in 2019 Q1 is unequivocally identified based on both series. However, there are two non-negligible differences for the other recessions (as also discussed below, concerning the Bry and Boschan procedure). First, there were two mild recessions (dark grey shadowed areas in Fig. 3), which we could detect based on the BCI but remained unobserved based on the technical definition. In both cases there were separate quarters with negative growth rates of real GDP. Second, in the remaining five recessions—in the early 1990s, in 2001/2002, in 2003, and in 2012—the BCI points to a one- to two-quarter longer recession than the technical definition would suggest. Moreover, the BCI could have indicated the technical recession both in 2001/2002 and in 2012 by one to two quarters earlier.

Another possible drawback of our approach is that—when our Business Cycle Indicator is calculated—the identification of the turning points in the business cycle is based on a graphical inspection of the Indicator. This procedure has been sometimes judged as insufficient in the literature, most probably due to its apparent simplicity. At the same time, this procedure is easily understandable and reproducible. Moreover, it is very flexible to account for non-linearities of the cycle. This is a great advantage compared with the standard rule- or algorithm-based approaches. This issue is especially problematic for parametric procedures but applies to non-parametric methods as well.

This notwithstanding, we checked for consistency of our results with the original Bry and Boschan (1971) approach. Their non-parametric procedure is based on an algorithm of pattern recognition, aiming at identifying the alternation of regimes between decreases and increases in economic activity.

We apply the Bry and Boschan (1971) algorithm to the quarterly series of our BCI and of real GDP and follow the insights of Harding and Pagan (2002). Accordingly, we set the minimum phase length to be two quarters and the minimum cycle length to be five quarters.

The results confirm an almost perfect consistency between the algorithm-based and the graphical inspection procedure for our BCI. In some few cases, the algorithm-based approach tends to anticipate the turning-point definition by one quarter earlier. The most striking case is the 2012/13 recession. The algorithm identified the peak already in the third quarter of 2011, whereas the graphical inspection pointed to the peak of the cycle in the second quarter 2012. A similar conclusion follows from the analysis of the real GDP time series. This underlines the importance of a critical attitude with respect to rule- and algorithm-based procedures.

As discussed in the methodology section, still another issue which could potentially undermine our approach concerns the fact that the data series used to estimate our Business Cycle Indicator are non-stationary. This is most probably due to the underlying trend in the series. Non-stationarity might be a problem in the PCA framework, as it can lead to only a few components carrying similar factor loadings (Lansangan & Barrios, 2009).

**Fig. 4:**
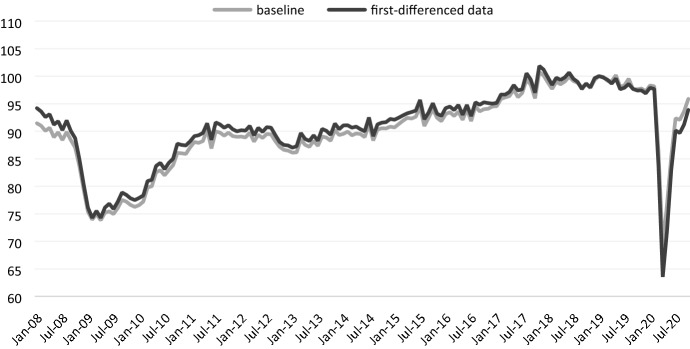
BCI in the baseline model versus BCI based on PCA on first-differenced data. Source: Own calculations Flossbach von Storch Research Institute/Macrobond

The analysis of the time-series properties of our data reveal that they are non-stationary in levels but stationary in first differences. To assess the influence of non-stationarity, we thus perform the PCA analysis on first-differenced data, recalculated our Business Cycle Indicator, and compared it with the baseline estimate as already shown in the previous section. Figure 4 summarizes our robustness check. Overall, there is a close correspondence between the baseline series and the new one. We can detect only a few differences between the two series, which, however, are contained and concern levels but not the underlying tendency.

## Conclusions and Outlook

Our analysis shows that the monthly series of the Business Cycle Indicator is a robust basis for dating the turning points of the German business cycle. It is also a good proxy for the development of real GDP. Compared to the existing rule of thumb, our Business Cycle Indicator has two advantages for the analysis of the business cycle. First, by assessing the information from a broad range of economic activity indicators it can more reliably identify business cycle peaks and troughs. Second, as it is based on monthly data it can more precisely monitor and time turning points of the business cycle. This has an advantage over quarterly GDP series, which not only are published with a delay, but are also often subject to subsequent revisions. Consequently, the BCI offers a useful, transparent and comprehensive tool of analysis for policy makers and practitioners continuously assessing the business cycle in Germany.

Based on our estimations, we could track the economic activity in Germany between 1991 and 2020. Regarding the most recent developments of the BCI, it stagnated from the second quarter of 2018 and started to decline in March 2019. Hence, we identified this month as the starting point of the most recent recession. Developments of the BCI for the rest of 2019 showed a substantial weakness of German economic growth. In seven months, the growth rates of the Indicator were negative, with months characterized by positive growth rates only weakly compensating for the BCI decline.

The subdued development of the economy during 2019 was subsequently overlapped by the detrimental impact of the COVID-19 pandemic, which drove the economy into a much deeper recession starting in March 2020. Since March 2019 we have, thus, experienced two overlapping patterns. The first recession period, between March 2019 and February 2020, is distinct from the pandemic. It is most likely driven by the negative impact of the ongoing trade disputes and deglobalization tendencies. The second period is unambiguously related to the current pandemic.

## Footnotes

1 Business cycle reference dates for the USA are computed back to 1857, but the official announcements started in 1979. For details, see https://www.nber.org/cycles.html.

2 For a detailed description of the NBER’s approach, see the press releases of the turning points in the business cycles, available at: https://www.nber.org/cycles.html.

3 “Determination of the December 2007 Peak in Economic Activity”, Business Cycle Dating Committee, National Bureau of Economic Research, December 1, 2008, available at https://www.nber.org/cycles/dec2008.html.

4 See “Annual report of the German Council of Economic Experts for 2008/2009”, p. 7, available at: https://web.archive.org/web/20090306123725/http://www.sachverstaendigenrat-wirtschaft.de:80/download/gutachten/ga08_i.pdf].

5 The origins of the term are unclear, but it is a largely accepted wisdom. In line with it, the UK’s economics & finance ministry (the Treasury) recognizes that “the commonly accepted definition of a recession in the UK is two or more consecutive quarters (a period of three months) of contraction in national GDP.”

6 To give an example, let’s compare two economies. The first experiences quarter-over-quarter growth rate of real GDP of − 2% in the first quarter and of − 4% in the third quarter, with zero growth in the other quarters of the year. The second faces negative growth of − 0.1% in the second and third quarter and positive growth in the first and fourth quarter. According to the conventional definition, only the latter economy was in recession, although the former suffers a more serious overall contraction.

7 The ECRI does not provide information either on the dataset or on the method used in the analysis. Moreover, updates of the chronology suffer from substantial lags of over one year. Also, the OECD publishes business cycle dates, determined through the Bry-Boschan algorithm applied on the OECD’s composite leading indicator on a quarterly rather than monthly basis.

8 There is still another related application of dynamic factor models, which, however, goes beyond the scope of this paper, given their focus on forecasting rather than on inspecting past business cycles. More specifically, this applies to approaches extracting information from a set of leading indicators, combing hard data with survey information. See, for example, the contributions by Camacho et al. (2014) and Carstensen et al. (2020).

9 The two approaches view economic fluctuations differently. Whereas the growth approach focuses on deviations of the activity from the long-term trend, the classical approach refers to fluctuations in the levels of economic activity. See Anas et al. (2008) for an exhaustive overview on the subject.

10 However, the Committee also determined that a peak in quarterly economic activity occurred in 2019 Q4, rather than in 2020 Q1, as the monthly dating would imply. This difference derives from the Committee’s policy of identifying the months and quarters of peak activity separately, without requiring that the monthly peak lies in the same quarter as the quarterly peak.

11 Following the NBER’s experience, the Centre for Economic Policy Research formed a dating committee in 2003 to set the turning points of the euro area business cycle. Their methodology is based, among others, on GDP figures and provides a quarterly chronology.

12 The method has been extensively used in different related contexts so far. It has gained popularity in the context of constructing different indices (Dreher, 2006; Gwartney and Lawson, 2001; Lockwood and Redoano, 2005). Also, other related applications are common. To give some examples, Inklaar et al. (2008) apply PCA to construct a single measure of trade intensity when studying business cycle synchronization in OECD countries. Their combined measure is the largest eigenvalue obtained from the common variation in six individual trade intensity measures and accounts for 64% of the total variance. König and Ohr (2013) rely on the PCA method to construct their EU Index, measuring the degree of integration between member states of the European Union.

13 It should be acknowledged, however, that the timing procedure based on the raw index data is not without problems, given the underlying volatility of the series. Accordingly, Zhao (2020) compares different smoothing methods, but emphasizes at the same time that—due to the aforementioned lag—“smoothing must be carefully performed to provide a balance between the smoothness and the lag” (Zhao, 2020, p.83).

14 We also adopted an alternative approach. First, we re-estimated the Principal Component Analysis for our sample of indicators on a quarterly basis for which data are available since 1991. Secondly, we checked in a simple regression analysis the extent to which the quarterly BCI has explanatory power for real GDP data. The results of this estimation—not reported here, but available upon request—are very much in line with the ones shown in Table 4, confirming the overall validity of our Indicator. However, the advantage of MIDAS compared with the traditional aggregation approach is that MIDAS exploits information from every observation in the higher frequency space.

15 Another valuable robustness check consists of estimating the quarterly BCI including real GDP data and checking how much of a difference this extension makes to our framework. The results, which we do not report here but are available upon request, indicate that the extension does not yield a significant change of the original series, despite a high factor loading of real GDP.

16 It is important to note that there are some crucial differences between the absolute values of the growth rates of the BCI and of GDP. The discrepancy is most likely driven by the fact that the underlying series of the BCI are more sensitive to short-term economic impulses and are thus more volatile than the GDP data. This constitutes a potential drawback, given that this increased sensitivity may go hand in hand with a higher risk of false recession signals. However, as pointed out in the methodology part of the paper, the adopted visual inspection approach requires for a recession to occur that the BCI shows a sustained decline followed by a sustained recovery. This should make sure that temporary signals are treated as noise.
